# Violence as a public health problem: An ecological study of 169 countries^☆^

**DOI:** 10.1016/j.socscimed.2013.12.006

**Published:** 2014-03

**Authors:** Achim Wolf, Ron Gray, Seena Fazel

**Affiliations:** a Department of Psychiatry, University of Oxford, UK; b National Perinatal Epidemiology Unit, University of Oxford, UK

**Keywords:** Crime, Violence, Public health, Income inequality, Alcohol

## Abstract

Individual level risk factors for violence have been widely studied, but little is known about country-level determinants, particularly in low and middle-income countries. We hypothesized that income inequality, through its detrimental effects on social cohesion, would be related to an increase in violence worldwide, and in low and middle-income countries in particular. We examined country-level associations of violence with socio-economic and health-related factors, using crime statistics from the United Nations Office on Drugs and Crime, and indicators from the Human Development Report published by the United Nations Development Programme. Using regression models, we measured relationships between country-level factors (age, education, measures of income, health expenditure, and alcohol consumption) and four violent outcomes (including measures of violence-related mortality and morbidity) in up to 169 countries. We stratified our analyses comparing high with low and middle-income countries, and analysed longitudinal data on homicide and income inequality in high-income countries. In low and middle-income countries, income inequality was related to homicide, robbery, and self-reported assault (all *p*'s < 0.05). In high-income countries, urbanicity was significantly associated with official assault (*p* = 0.002, *β* = 0.716) and robbery (*p* = 0.011, *β* = 0.587) rates; income inequality was related to homicide (*p* = 0.006, *β* = 0.670) and self-reported assault (*p* = 0.020, *β* = 0.563), and longitudinally with homicide (*p* = 0.021). Worldwide, alcohol consumption was associated with self-reported assault rates (*p* < 0.001, *β* = 0.369) suggesting public policy interventions reducing alcohol consumption may contribute to reducing violence rates. Our main finding was that income inequality was related to violence in low and middle-income countries. Public health should advocate for global action to moderate income inequality to reduce the global health burden of violence.

## Highlights

• In low and middle-income countries, income inequality is related to rates of homicide and assault.
• In high-income countries, urbanicity is associated with assault and robbery rates.
• In high-income countries, income inequality is related to homicide and self-reported assault rates.
• Worldwide, alcohol consumption is associated with self-reported assault rates.

## Introduction

There is increasing recognition of the public health importance of violence (Krug, Mercy, Dahlberg, & Zwi, 2002). It is currently among the top twenty causes of worldwide loss of disability-adjusted living years, and projected to increase in importance by 2030 according to the World Health Organization (Mathers, Fat, & Boerma, 2008). Consequently its impact on health services is substantial, costing $5.6 billion a year in the US alone (Corso, Mercy, Simon, Finkelstein, & Miller, 2007). In particular, acute medicine, surgery, and psychiatry spend considerable sums treating victims of violence. Head injury, for example, is currently the commonest presentation to emergency departments in some western countries (Bruns & Hauser, 2003). Violence against women is a serious public health issue (Campbell, 2002; Garcia-Moreno, Jansen, Ellsberg, Heise, & Watts, 2006) and associated with poor maternal and foetal outcomes (Garcia-Moreno, 2009). Particularly in urban areas, fear of violence undermines people's health and wellbeing (Kjellstrom et al., 2007).

Despite the importance of violence to health outcomes, the social and economic determinants of violence need further clarification. Individual-level factors have been investigated in longitudinal cohorts and population-based studies. Several characteristics are predictive of violent offending: behavioural problems at a young age, including aggression and under-age smoking and drinking (Loeber & Farrington, 1998; West & Farrington, 1973); low IQ and educational achievement (Farrington & Welsh, 2007); low family income (Loeber & Farrington, 1998); male gender (Moffitt, 2001). Age is strongly related to criminal behaviour, and the prevalence of violence increases almost 10-fold during adolescence (Moffitt, 1993). However, less is known about neighbourhood and country-level factors.

Theoretical models have examined the role of income and societal inequalities as factors directly related to violence. For instance, in Merton's theory of anomie (Merton, 1938), violent crime and other forms of ‘rebellion’ are seen as a mode of adaption to counter society's emphasis towards success goals in persons who cannot access the means to achieve these goals. This theory has parallels with the notion of relative deprivation in criminology, which suggests that such discontent motivates or disinhibits individuals to engage in violent acts (Patterson, 1991) and that ecological socioeconomic factors could therefore be closely linked to crime rates (Shaw & McKay, 1942). Researchers who have focused on structural violence discuss how social structures, through factors such as poverty, discrimination, and socio-economic inequality may systematically disadvantage certain groups of individuals in society (Farmer et al., 2004). Structural violence is generally ‘invisible’ and embedded in “ubiquitous social structures, normalized by stable institutions and regular experience” (Gilligan, 1997, 306). However, it has been suggested that structural violence and interpersonal violence are closely related (Blau & Blau, 1982). More recently, it has been theorized that relative deprivation causes stress through a decreased sense of self-worth and a lack of autonomy, which in turn leads to violence (Marmot, 2004; Wilkinson, 2006). Alternative theories of crime focus on the individual and, in particular rational choice theory, argue that a criminal act is the outcome of an internal deliberation and weighing up the positive and negative consequences of the crime (Cornish & Clarke, 1986). However, while this has a strong face validity for ‘instrumental’ crimes such as theft and burglary, ‘expressive’ crimes (including many homicides) involve emotion and a disregard for future consequences (Salfati, 2000), and therefore rational choice theory may not be as relevant.

The theoretical effects of income inequality on crime have been given some empirical support. Preliminary work has been conducted on country-level factors, and a number of potentially important findings have been published. Wilkinson and Pickett have found evidence that homicide is associated with income inequality (as measured by metrics such as the Gini index) in 23 countries (Pickett, Mookherjee, & Wilkinson, 2005; Wilkinson & Pickett, 2006, 2009). However, this work is based on high-income countries only, was based on data up to 2000, and examined solely homicide as an outcome. A 2004 systematic review of the effects of income inequality on population health supports their homicide finding for high-income countries (Lynch et al., 2004). More recent work has looked at possible mediators of the relationship between income equality and homicide, although again the number of countries has remained restricted (Elgar & Aitken, 2011; Nadanovsky & Cunha-Cruz, 2009; Nivette, 2011).

Furthermore, from a public health perspective, assault is an important measure of violence-related morbidity with rates on average over twenty times higher than for homicides (UNODC, 2011). Therefore in this study we have looked at a broader range of violent outcomes (homicide, self-reported assault, official assault, robbery), examining countries irrespective of income levels, using multivariate and longitudinal analyses. These outcomes are complementary as they represent measures of violence-related mortality (homicide) and morbidity (assault and robbery). We hypothesized that income inequality would be independently associated with violent outcomes in all countries, not solely high-income ones.

## Methods

We measured the strength of the relationships between a range of socioeconomic factors and various violent outcomes rates across 169 countries. We conducted subgroup analyses on OECD countries (Organisation for Economic Co-operation and Development, *n* = 34) and countries in the MPI (Multidimensional Poverty Index, *n* = 94, excluding the six countries that are also in the OECD), to compare factors affecting violent outcomes between high-income, and low and middle-income countries, respectively. We repeated our analyses using the countries in the studies by Wilkinson and Pickett that contributed to their book *The Spirit Level* (Wilkinson & Pickett, 2009), and related empirical studies (Pickett, Kelly, Brunner, Lobstein, & Wilkinson, 2005; Pickett, Mookherjee, et al., 2005; Wilkinson & Pickett, 2006), allowing us to examine whether their bivariate analyses and our regression models come to similar conclusions. This sample (*n* = 23) was chosen from the richest 50 countries excluding those with populations of less than three million and those without UN income data (Wilkinson & Pickett, 2009). We included MPI poverty data in our model to establish whether levels of absolute poverty could in part explain differences in violent outcomes. Finally, we examined the relationship of income inequality on homicide rates over time in OECD countries (230 data points in 27 countries).

## Country data

The following variables were included in our primary models: 1) Gross National Income (GNI), which has been shown to have bidirectional relationships with violent crime (Bourguignon, 1999); 2) Years of schooling, as higher education levels are associated with lower crime (Lochner & Moretti, 2001); 3) Income inequality, as it is associated with a range of adverse population health outcomes (Nivette, 2011; Wilkinson & Pickett, 2006); 4) Labour force participation, as employment is related to lower rates of crime (Sherman, Farrington, Welsh, & MacKenzie, 2002); 5) Urbanicity, as there is evidence that violence rates are higher in cities (Glaeser & Sacerdote, 1996); 6) Median age, as there is a well-established relationship between younger age and crime (Farrington, 1986); 7) Health expenditure, as poor health was found to be associated with crime in a study of South London males (Farrington, 1995); and 8) Alcohol consumption, because of large numbers of crimes being alcohol-related (Greenfeld, 1998), and a recent meta-analysis reported strong associations with violence (Wagenaar, Tobler, & Komro, 2010). This meta-analysis estimated the standardized effect sizes of alcohol tax and price policies on violence at −0.022 for violence (95% confidence interval: −0.034 to −0.010), and −0.014 for crime (−0.023 to −0.005). The review concludes that a doubling of alcohol taxes in the US would be associated with a 2% reduction in violence and 1.2% reduction in crime (Wagenaar et al., 2010). The effects of gun ownership, as more limited data was available, were analysed in a sensitivity analysis. Drug consumption was not included in our final models due to a significant proportion of missing data, particularly in low and middle-income countries.

Data were taken primarily from the Human Development Report published by the United Nations Development Programme (UNDP, 2010). The UNDP tables were used for homicide (per 100,000 people, 2003–2008) and robbery rates (per 100,000 people, 2003–2008), and self-reported assault (% reporting having been a victim, 2006–2009). Official assault rates (per 100,000 people, 2003–2008) and longitudinal homicide statistics (per 100,000 people, 1995–2009) came directly from the UNODC Crime & Criminal Justice Statistics (UNODC, 2011). Time points varied based on country availability, and averages of all recorded data points were used in our model. All UN crime data was compiled from police, prosecution, court, and prison authorities. Self-reported assault rates come from the Gallup World Poll database, which posed the question “Within the past 12 months, have you been assaulted or mugged?” (UNDP, 2010), typically using a representative sample of 1000 completed questionnaires per country. Their methods are described in more detail elsewhere (Gallup, 2007), although data on the validity and reliability of this questionnaire is not available. Robbery was included as it is a violent crime (unlike theft) and leads to physical and psychological morbidity (Cook, 1987). Homicide, official assault, and robbery were transformed into “per 100,000 people aged 15–64,” using the dependency ratio data from the UNDP Statistical Tables, to account for differences in age structures.

Data on the following were also taken from the UNDP: Gross National Income (GNI) per capita (Purchasing Power Parity [PPP] in 2008 US$, 2010), mean years of schooling (2010), income Gini coefficient (2000–2010), labour force participation rate (%, 2008), urbanicity (% of total, 2010), median age (2010), expenditure on health per capita (PPP in US$, 2007) and Multidimensional Poverty Index (2000–2008). Longitudinal income Gini coefficient data (1995–2009) came from the OECD Income distribution and poverty database and was available for 27 countries. The Gini coefficient is a measure of income inequality ranging from 0 to 100; 0 represents perfect equality and 1 perfect inequality. World Health Organization data were used for alcohol use (total adult per capita consumption, 2005) (World Health Organization, 2012), and are based on data primarily from government sources and country-specific alcohol industry statistics, and from the Food and Agriculture Organization of the United Nations' statistical database (FAOSTAT). Civilian firearm ownership data (guns per 100 people) were taken from the 2007 Small Arms Survey (Karp, 2007).

All data sources and definitions are shown in table 1.

## Analyses

We fitted complete-case linear regression models, separately for the four outcome variables (rates of homicide, self-reported assault, official assault, and robbery). Due to theoretical justification for inclusion, covariates were included in the model regardless of statistical significance (Rothman, Greenland, & Lash, 2008). Regression models were fitted separately for two subgroups: OECD and MPI countries, roughly encompassing high-income, and middle and low-income countries, respectively. The Czech Republic, Estonia, Hungary, Slovakia, Turkey, and Mexico appeared in both the OECD and MPI groups and were therefore excluded from analyses of the latter to obtain distinct groups.

We repeated our regression analyses restricting our dataset to the countries in the Wilkinson and Pickett studies (*n* = 23) to compare our results with theirs. We conducted a sensitivity analysis for our homicide model, excluding (less reliable) public health estimates for homicides, looking only at criminal justice data. We fitted our models again, excluding countries with on-going conflicts (defined as 1000+ deaths per year) (Uppsala Universitet, 2012). We also repeated our homicide analyses, including data on gun ownership and compared this to the more parsimonious models.

In each model, we examined Variance Inflation Factors (VIF) for multicollinearity; VIF > 10 being used as a cut off value (Neter, Wasserman, & Kutner, 1989). When pairs of variables caused multicollinearity, the variable with fewer missing data was excluded from the model, and significant differences were reported. As a result, we repeated all OECD and one MPI stratified analyses excluding the health expenditure variable (VIF > 10).

For our longitudinal analysis of homicide we fitted a complete-case linear regression model, using Gini as a predictor, adjusting for year of measurement, and using Stata's *vce(cluster)* command to obtain a Huber-White robust variance estimate that adjusts for within-country correlation (Rogers, 1993), as used in related work (Mobley et al., 2006; Weich, Lewis, & Jenkins, 2002).

Multivariate analyses were conducted using SPSS 20 (IBM, 2011). The longitudinal analysis was conducted using Stata 12 (StataCorp, 2011).

## Results

**Fig. 1:**
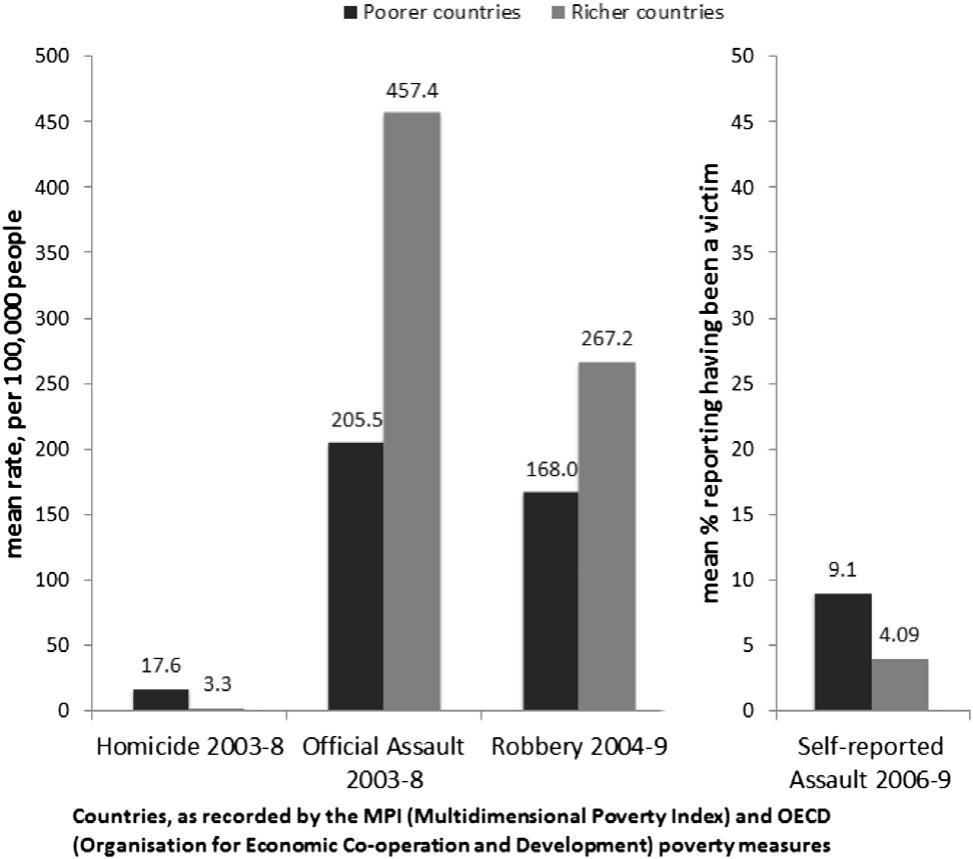
Violent outcome rates, by country type.

The overall regression models included 140 countries for homicide, 128 for self-reported assault, 80 for official assault, and 91 countries for robbery (Table 2). Homicide and self-reported assault rates were generally higher in low and middle-income countries (Fig. 1). Typically, 9.1% reported having been a victim of assault in the last 12 months in low and middle-income countries, compared to 4.1% in high-income countries. As expected, there were significant differences in average socio-economic characteristics between MPI and OECD countries (Table 3). Official and self-reported assaults were significantly correlated in MPI (*r* = 0.536, *p* = 0.001) but not OECD countries (*r* = 0.160, *p* = 0.366). The longitudinal analysis included 230 data points for 27 countries from 1995 to 2009 (between 3 and 15 data points per country; median 8 data points per country).

### Homicide

In our general model, there was evidence for a positive relationship between homicide rates and Gini (*β* = 0.344, *p* < 0.001), and an inverse relationship between homicide rates and median age (*β* = −0.361, *p* = 0.049; inverse relationship). After stratifying by OECD and MPI poverty measures, Gini was also significant in the OECD group (*β* = 0.670, *p* = 0.006). In the MPI countries, median age (*β* = −0.538, *p* = 0.009; inverse relationship), and Gini (*β* = 0.334, *p* = 0.004) were significant.

In bivariate analyses, gun ownership was negatively associated with homicide rates worldwide (*p* = 0.036), but not in MPI (*p* = 0.479) or OECD subsamples (*p* = 0.886). Inclusion of the variable in our multivariate models did not materially alter our results; gun ownership was not significantly associated with homicide rates in any of the three models (data not shown).

In our longitudinal model, Gini was positively associated with homicide rates (coefficient: 24.2, 95% confidence interval: 4.0 to 44.4, *p* = 0.021).

### Self-reported assault

Median age (*β* = −0.544, *p* < 0.001; inverse relationship), alcohol consumption (*β* = 0.369, *p* < 0.001), Gini (*β* = 0.345, *p* < 0.001), urbanicity (*β* = 0.277, *p* = 0.005), and years of schooling (*β* = −0.227, *p* = 0.015; inverse relationship) were all significant in explaining self-reported assault rates in all included countries. Gini was significant in the OECD sample (*β* = 0.563, *p* = 0.020). In the MPI sample, median age (*β* = −0.424, *p* = 0.020; inverse relationship), Gini (*β* = 0.349, *p* < 0.001), alcohol consumption (*β* = 0.252, *p* = 0.038), and urbanicity (*β* = 0.251, *p* = 0.027) were all significant.

### Official assault

Overall, and in the MPI sample, we found no significant relationships. Urbanicity (*β* = 0.716, *p* = 0.002) was significant in the OECD sample.

### Robbery

Overall, Gini (*β* = 0.341, *p* = 0.014) was associated with robbery rates. In the OECD sample, only urbanicity (*β* = 0.587, *p* = 0.011) was significant. In the MPI sample, both median age (*β* = −0.577, *p* = 0.027; inverse relationship), and Gini (*β* = 0.307, *p* = 0.046) were significant.

## Further analyses

Using the Wilkinson and Pickett countries (*n* = 23), we found no significant associations for homicide and self-reported assault. Urbanicity was the only significant predictor for official assault (*β* = 0.611, *p* = 0.041) and robbery (*β* = 0.608, *p* = 0.035) rates.

In bivariate analyses, drug consumption was not significantly correlated with any violent outcomes in the whole sample of countries (data not shown). Similarly, the relationship was non-significant when included in our regression models. We found multicollinearity (VIF > 10) between GNI and health expenditure for all four outcomes in the OECD sample, and between the same two variables for official assault rates in the MPI sample. When we re-ran our models, excluding the health expenditure variable, Gini was no longer significantly associated with self-reported assault in the OECD sample (from *p* = 0.020 to *p* = 0.059). There were no other material changes to our results.

Restricting our overall homicide model to countries for which criminal data (as opposed to public health data) were available to determine homicide rates changed the significance of the median age variable (from *p* = 0.049 to *p* = 0.051) but had no significant impact on Gini or other variable associations. Excluding countries (*n* = 6) with on-going military conflicts did not materially alter our results (results available on request).

## Discussion

In a study of 169 countries from 2003 onwards, we looked at associations between four violent outcomes and a range of socioeconomic factors. The results support previous work underlining the large public health burden of violence, particularly in terms of violence-related morbidity in low and middle-income countries. We found that typically 9% of individuals in low and middle-income countries reported being the victim of an assault in the previous year. Our analyses demonstrated that in high-income countries, urbanicity was associated with official assault and robbery rates, and income inequality was associated with homicide and self-reported assault. The relationship between income inequality and homicide was also confirmed in within-country models from 1995 to 2009. In low and middle-income countries, income inequality was related to homicide, self-reported assault, and robbery. In addition, we found that alcohol consumption was associated with self-reported assault rates worldwide, an index of violence-related morbidity. Our results are compatible with the hypothesis that high crime rates are a consequence of a breakdown of social cohesion, which is associated with, and potentially caused by, large differences in opportunity and income levels (Kawachi, Kennedy, Lochner, & Prothrow-Stith, 1997).

Three main implications follow from these findings for public health and policy. First, public health should advocate for governmental measures to reduce income inequality, such as labour market reforms, distributive fiscal policy, and expansion of financial markets for small enterprises (World Bank, 2000). Absolute poverty also needs to be tackled, and a recent study concluded that rises in unemployment are associated with increased rates of homicide (Stuckler, Basu, Suhrcke, Coutts, & McKee, 2009). Second, our results further strengthen the case for restricting alcohol supply by pricing (Wagenaar, Salois, & Komro, 2009; Wagenaar et al., 2010), reducing availability and limiting marketing to children (Chisholm, Doran, Shibuya, & Rehm, 2006), and other measures to reduce societal violence. Targets for reductions in alcohol-driven violence could also be considered as part of any national alcohol strategy (Sheron, Gilmore, Parsons, Hawkey, & Rhodes, 2012). However, while a substantial body of evidence exists in support of a range of alcohol policy interventions, implementation faces many challenges worldwide. Currently, policy options considered by many governments are influenced by alcohol industry interests and include ineffective strategies such as education around health effects, and alcohol is treated as an ordinary, non-hazardous, commodity (Room, Babor, & Rehm, 2005). Finally, legislative changes may have a role. Restrictions on gun ownership, allied with other measures, are likely to reduce gun crime and homicide rates in some countries (The Lancet, 2007). The contribution of legislation in reducing violence-related mortality and morbidity towards women and children needs further examination (Reichenheim et al., 2011).

Our findings on homicide in high-income countries support previous empirical research in the field (Lynch et al., 2004; Pickett, Mookherjee, et al., 2005; Wilkinson & Pickett, 2006, 2009) and theoretical explanations based on anomie theory (Savolainen, 2000). In relation to other outcomes, previous work has examined robbery and homicide data up to 1995 in 39 countries and found a positive correlation between crime and income inequality (Fajnzlber, Lederman, & Loayza, 2002). We found this association between robbery and inequality worldwide, but when stratifying by country type, this was not present in high-income countries. To our knowledge, no previous research has systematically examined country-level associations with official or self-reported assault.

Previous work has posited that relative deprivation leads to a lack of social cohesion and thus violence (Shaw & McKay, 1942) and, more recently, that stress explains the association of income inequality and a range of poor health outcomes such as life expectancy, obesity, teenage pregnancy, infant mortality, and violence (Wilkinson & Pickett, 2006, 2009). We found that income inequality was strongly associated with robbery in low and middle-income countries, but not in high-income OECD countries. On the basis of this, we would argue that our findings on robbery are broadly consistent with anomie theory, and specifically with a justification hypothesis of violence (that is, individuals in countries with more extreme income inequality may feel more justification to rob others) (Hayward & Young, 2007) or that such inequality contributes to a disruption of social relationships that in turn lead to crime and violence (Fukuyama, 1999). Specifically, anomie theory suggests that large differences in economic resources lead to resentment and animosity towards others, which in turn can result in criminal and violent behaviour (Jacobs, 1981). Our findings on the relationship between alcohol and crime hold for self-reported assault rates, but not for police-reported or official ones. This may be a reflection of the fact that domestic violence is both more associated with alcohol (Gilchrist et al., 2003), and less likely to be reported to the police (Abramsky et al., 2011). Furthermore, previous research has found little support that anomie is directly related to drinking habits (Johnson & Matre, 1978). We found one socioeconomic factor was associated with two violent outcomes in OECD countries, namely urbanicity with official assault and robbery rates. This is not surprising as rates for these crimes will be influenced by opportunities to commit them, but also secondary to high-income countries having better methods of detection. Whether increasing urbanization needs to be associated with differential allocation of police and crime prevention resources will require more research. The urbanization finding is partly in contrast to studies within countries that have found that absolute and fatality rates for accidents are mostly higher for rural populations (Peek-Asa, Zwerling, & Stallones, 2004). The association between a younger population and violent outcomes underlines the contribution of young people to violent crime, and school-based primary and secondary prevention interventions should be considered. A review of secondary violence prevention in schools has found that interventions, in particular those designed to improve relationship or social skills, can reduce aggressive behaviour (Mytton, DiGuiseppi, Gough, Taylor, & Logan, 2006). A separate review found that family/parent training programs are effective in reducing antisocial and delinquent behaviour in schools (Piquero, Farrington, Welsh, Tremblay, & Jennings, 2009). Whether these findings translate to low and middle-income countries needs further research.

### Strengths and limitations

We used a large sample of high, medium and low-income countries worldwide. To our knowledge, this is the largest country-level study to date on the association between violent outcomes and social and economic determinants. Unlike previous studies on inequality and crime (Elgar & Aitken, 2011; Fajnzlber et al., 2002; Pickett, Mookherjee, et al., 2005), we have included other socioeconomic factors in our models and the use of homicide, self-reported assault, official assault, and robbery rates, allowed us to contrast the importance of variables by outcome, and to test for consistency across country-types.

However, there are some important limitations to this study. First, our focus on a specific range of socioeconomic and public health factors was partly restricted by the availability in the Human Development Reports and other international databases, and we were unable to examine some previously researched factors such as trust (Elgar & Aitken, 2011), or ethnic heterogeneity (Cole & Gramajo, 2009), due to these data being available for a restricted number of countries only. Second, limitations of ecological cross-sectional designs mean that inferences on both within-country and individual differences, and causality, should be made with considerable caution. We attempted to address this limitation by examining homicide and income inequality data longitudinally in a sample of countries. Confidence intervals in our longitudinal analysis were wide, so while the direction and significance of this relationship appear robust, caution is advised when interpreting its magnitude. Although longitudinal work in Australia has found a significant reduction in firearm homicides and mass shootings in the decade after gun ownership restrictions were introduced in 1996 (Chapman, Alpers, Agho, & Jones, 2006), our bivariate cross-sectional findings on gun ownership are likely to be confounded as gun ownership was associated with higher income, which itself was associated with lower homicide levels. Thus, the association disappeared when stratifying by country-type, and in multivariate models. Finally, further work needs to examine the validity of the violent outcome measures used here, particularly in relation to assault, and data quality for all country-level factors and outcomes in low and middle-income countries. Furthermore, data was unavailable for certain low and middle-income countries and violent outcomes, and future work could examine whether the lack of data is related to a more limited criminal justice system, and/or higher rates of violent crime.

While the UNDP justifies indicators' inclusion as based on their “conceptual relevance, accuracy and reliability, consistency, and availability” (Kovacevic, 2010, 7), data collection has not been systematically evaluated for reliability, and cross-country comparisons may be poor. Critics have questioned the ability of HDI indicators to discriminate between countries, in particular at the top and bottom ends of the distribution (Anand & Sen, 1994). Furthermore, assaults and robberies may be less likely to be officially recorded in countries with limited judicial or police structures. This means that caution is warranted in interpreting the findings on robbery and assault in relation to middle and low-income countries. Our sensitivity analysis on high-income countries alone addresses part of this limitation. Homicide data, however, is less subject to this shortcoming as both criminal justice data (national government sources) and public health data (cause of death data, not dependent on conviction) are used (UNODC, 2011). Differences in self-reported assault in the Gallup survey may partially reflect cultural differences and country-specific experiences of assault, in particular when comparing high and low-income countries. Our finding that rates of self-reported assault are related to official assault in low and middle-income countries, but not high-income countries underlines this. Further research on the validity of self-reported assault measures, and their potential relationship to both unreported domestic violence, and general feelings of personal security, is warranted. While the illicit gun trade may underestimate our gun ownership variable, the Small Arms Survey utilizes data on unregistered ownership using import/export statistics, and independent estimates. Alcohol has been associated with violent crime, and with domestic violence in particular (Abramsky et al., 2011); in addition to the direct effects of alcohol on violence, inequality could lead to higher consumption, which in turn leads to further increased rates of violence. Abuse of alcohol could be modifier of the effects of inequalities on violence. As domestic violence is often underreported, this could explain our findings that alcohol consumption is related to self-reported, but not official assault rates.

In summary, we have found that income inequality as measured by the Gini is associated with certain violent outcomes in both high, and low and middle-income countries, and alcohol consumption with self-reported assault rates in all countries. We also found that urbanicity was associated with official assault and robbery rates in high-income countries. The role of public policy in reducing the health burden of violence needs further examination.

## Figures and Tables

**Table 1:** Data sources and definitions.

Variable	Source	Definition	Time period
Homicide	United Nations Office on Drugs and Crime	Official homicide rates (per 100,000), 2003–2008	2003–2008
Official assault	United Nations Office on Drugs and Crime	Official assault rate (per 100,000)	2003–2008
Self-reported assault	United Nations Development Programme	Self-reported assault (% reporting having been a victim)	2006–2009
Robbery	United Nations Office on Drugs and Crime	Robbery rate (per 100,000). Property crime involving violence or threat of violence.	2004–2009
GNI	United Nations Development Programme	Gross National Income, per capita (PPP US$ 2008)	2010
Years of schooling	United Nations Development Programme	Mean years of schooling	2010
Gini^3^	United Nations Development Programme	Income Coefficient, 0 (perfect equality) to 100 (perfect inequality)	2000–2010
Labour force part.^4^	United Nations Development Programme	Total participation rate (%)	2008
Urbanicity^5^	United Nations Development Programme	% Of total	2010
Age^6^	United Nations Development Programme	Median age	2010
Health expenditure^7^	United Nations Development Programme	Per capita (PPP US$)	2007
Alcohol^8^	World Health Organization	Total adult consumption	2005
MPI^9^	United Nations Development Programme	Multidimensional poverty index (Health; Education; Living standards)	2000–2008
Guns	Small Arms Survey 2007	Civilian firearm ownership data (guns per 100 people)	1998–2008

**Table 2:** Associations between country-level socioeconomic factors and violent outcomes.

		Overall *n* = 140		MPI^i^ countries *n* = 88		OECD^j^ countries *n* = 32	
		Beta	*p*	Beta	*p*	Beta	*p*
Homicide rate (per 100,000), 2003–2008	GNI^a^	0.032	0.876	0.224	0.418	0.088	0.834
	Years of schooling^b^	0.203	0.136	0.090	0.675	0.072	0.639
	Gini^c^	0.344	<0.001**	0.334	0.004**	0.670	0.006**
	Labour force part.^d^	−0.011	0.894	0.034	0.759	0.138	0.497
	Urbanicity^e^	−0.075	0.520	−0.233	0.070	0.148	0.343
	Age^f^	−0.361	0.049*	−0.538	0.009**	−0.307	0.144
	Health expenditure^g^	−0.029	0.881	0.150	0.600	−0.234	0.482
	Alcohol^h^	0.077	0.491	0.038	0.785	0.251	0.195
	MPI^i^			−0.212	0.408		
	Intercept	1.74		25.54		−11.53	
	Adjusted *r*-squared	0.198		0.189		0.554	

		Overall *n* = 80		MPI^i^ countries *n* = 36		OECD^j^ countries *n* = 32	
		Beta	*p*	Beta	*p*	Beta	*p*
Official assault rate (per 100,000), 2003–2008	GNI^a^	−0.100	0.723	0.622	0.324	0.204	0.712
	Years of schooling^b^	0.030	0.865	0.571	0.186	−0.290	0.163
	Gini^c^	0.152	0.328	0.387	0.099	−0.038	0.896
	Labour force part.^d^	−0.037	0.774	−0.230	0.255	−0.014	0.960
	Urbanicity^e^	0.082	0.639	−0.349	0.142	0.716	0.002**
	Age^f^	−0.234	0.340	−0.471	0.199	0.031	0.909
	Health expenditure^g^	0.503	0.055	0.015	0.979	−0.112	0.798
	Alcohol^h^	0.090	0.577	0.068	0.788	0.170	0.500
	MPI^i^			0.532	0.311		
	Intercept	178.66		245.22		−932.44	
	Adjusted *r*-squared	0.041		0.089		0.217	

		Overall *n* = 128		MPI^i^ countries *n* = 82		OECD^j^ countries *n* = 32	
		Beta	*p*	Beta	*p*	Beta	*p*
Self-reported assault (% reporting having been a victim), 2006–2009	GNI^a^	−0.019	0.912	0.034	0.887	0.372	0.388
	Years of schooling^b^	−0.277	0.015*	−0.057	0.760	−0.195	0.224
	Gini^c^	0.345	<0.001**	0.349	<0.001**	0.563	0.020*
	Labour force part.^d^	0.072	0.307	0.071	0.458	−0.189	0.367
	Urbanicity^e^	0.277	0.005**	0.251	0.027*	0.301	0.068
	Age^f^	−0.544	<0.001**	−0.424	0.020*	−0.091	0.666
	Health expenditure^g^	−0.004	0.982	0.114	0.636	−0.496	0.153
	Alcohol^h^	0.369	<0.001**	0.252	0.038*	0.320	0.111
	MPI^i^			0.270	0.227		
	Intercept	2.62		−3.67		−0.42	
	Adjusted *r*-squared	0.509		0.447		0.530	

		Overall *n* = 91		MPI^i^ countries *n* = 45		OECD^j^ countries *n* = 32	
		Beta	*p*	Beta	*p*	Beta	*p*
Robbery rate (per 100,000), 2004–2009	GNI^a^	−0.202	0.415	0.089	0.779	0.377	0.516
	Years of schooling^b^	−0.061	0.692	0.162	0.565	−0.178	0.409
	Gini^c^	0.341	0.014*	0.307	0.046*	0.373	0.233
	Labour force part.^d^	−0.113	0.320	−0.009	0.952	−0.393	0.171
	Urbanicity^e^	0.293	0.054	0.143	0.354	0.587	0.011*
	Age^f^	0.019	0.933	−0.577	0.027*	0.306	0.289
	Health expenditure^g^	0.282	0.223	0.668	0.053	−0.207	0.653
	Alcohol^h^	0.145	0.348	0.009	0.962	0.360	0.181
	MPI^i^			−0.019	0.954		
	Intercept	−345.19		−39.54		−2474.31	
	Adjusted *r*-squared	0.115		0.409		0.141	

Notes: * = *p* values less than 0.05; ** = *p* values less than 0.01; beta = standardized coefficient.

a Gross National Income, per capita (PPP US$ 2008), 2010.

b Mean years, 2010.

c Income Coefficient, 2000–2010.

d Total participation rate (%), 2008.

e % of total, 2010.

f 2010.

g Per capita (PPP US$), 2007.

h Total adult consumption, 2005.

i Multidimensional Poverty Index, 2000–2008 (excluding 6 countries also in the OECD sample).

j Countries in the Organisation for Economic Co-operation and Development.

**Table 3:** Median and standard deviation of violent outcomes and socioeconomic variables.

	Overall		MPI		OECD	
	Median	SD	Median	SD	Median	SD
Homicide^a^	5.4	20.0	9.3	20.8	2.2	3.6
Official assault^b^	124.0	387.3	54.3	399.8	311.5	398.8
Self-reported assault^c^	6.0	6.5	8.0	7.1	3.0	2.7
Robbery^d^	69.3	368.4	60.7	289.6	82.0	541.2
GNI^e^	7258.0	15,772.3	3280.0	7071.0	31,369.5	10,663.4
Years of schooling^f^	8.0	3.0	6.5	2.8	10.6	1.4
Gini^g^	39.8	9.5	42.5	9.4	33.4	6.7
Labour force part.^h^	70.1	9.1	70.9	9.5	73.1	7.3
Urbanicity^i^	58.2	23.1	48.2	19.5	78.6	11.6
Age^j^	26.3	8.5	22.2	6.7	39.9	4.2
Health expenditure^k^	402.0	1290.9	143.0	322.6	2783.5	1423.4
Alcohol^l^	6.1	4.6	5.2	4.0	10.9	3.5

a Homicide rate (per 100,000), 2003–2008.

b Official assault rate (per 100,000), 2003–2008.

c Self-reported assault (% reporting having been a victim), 2006–2009.

d Robbery rate (per 100,000), 2004–2009.

e Gross National Income, per capita (PPP US$ 2008), 2010.

f Mean years, 2010.

g Income Coefficient, 2000–2010.

h Total participation rate (%), 2008.

i % of total, 2010.

j 2010.

k per capita (PPP US$), 2007.

l Total adult consumption, 2005.
